# The Impact of Luting Agents on the Pulp of Vital Teeth: A Systematic Review

**DOI:** 10.3390/dj14070442

**Published:** 2026-07-15

**Authors:** Georgia Andreou, Maria Dede, Ioanna Pouliezou, Nikolaos P. Kerezoudis

**Affiliations:** 1 Department of Endodontics, School of Dentistry, National and Kapodistrian University of Athens, 2 Thivon Str., 115 27 Athens, Greece; mdede@dent.uoa.gr (M.D.); nkerez@dent.uoa.gr (N.P.K.); 2 Department of Orthodontics, School of Dentistry, National and Kapodistrian University of Athens, 2 Thivon Str., 115 27 Athens, Greece; ipouliez@dent.uoa.gr

**Keywords:** luting cements, dental cements, cementation, pulp vitality, pulp response, dental pulp

## Abstract

**Background/Objectives**: Luting agents are used to cement indirect restorations to teeth, and some of their components may induce pulpal reactions. This systematic review aims to evaluate the biocompatibility and effects of contemporary luting cements on the vital pulp. **Methods**: A comprehensive review of electronic databases PubMed, Cochrane Library, ScienceDirect, Web of Science, Scopus, and ClinicalTrials.gov was conducted for studies published between 2000 and 2026. Search strategies included MeSH terms, Boolean operators, and controlled vocabulary. Eligible studies included in vitro, ex vivo, in vivo, and clinical trials assessing pulpal responses to definitive luting cements in vital teeth or human-relevant pulp cell models. Screening and data extraction were performed independently by two reviewers. Risk of bias was assessed using the RoB 2 tool for clinical studies, QUIN for in vitro studies, SYRCLE for animal studies, and ROBINS-I for non-randomized human in vivo designs. Due to heterogeneity, meta-analysis was not performed, and a narrative synthesis was conducted. **Results**: From 1514 records, 40 studies met the inclusion criteria. Evidence was heterogeneous across study designs and outcomes. Human in vivo studies showed that short-term pulpal inflammation decreased over time, influenced by the remaining dentin thickness and material type. Clinical studies reported variable postoperative sensitivity. In vitro and ex vivo studies demonstrated material- and protocol-dependent effects on cell viability, oxidative stress, inflammatory markers, monomer release, and intrapulpal temperature rise. **Conclusions**: Luting cement selection should be case-dependent. Dual-cure, self-adhesive resin cements appear to demonstrate favorable biocompatibility in vital teeth; however, application protocols may influence cytotoxicity. Further clinically relevant studies are needed to support safer and biologically wise material selection. **Registration**: PROSPERO CRD420251156047.

## 1. Introduction

Luting agents are essential for the cementation of indirect restorations; however, their interaction with the dentin–pulp complex remains a biological concern [1]. Components of contemporary luting cements, particularly resin-based systems, may diffuse through dentin and interact with pulpal tissues, potentially inducing inflammatory responses, cytotoxic effects, or thermal injury [2]. These biological mechanisms are clinically relevant, as they may contribute to postoperative sensitivity and pulpal irritation following restorative procedures [3].

Studies have investigated pulpal responses using a wide range of experimental models, including in vitro cell cultures, ex vivo dentin barrier systems, animal histology, and clinical trials. However, the available evidence remains fragmented, with considerable variability in materials tested, experimental conditions, and outcome measures. In particular, discrepancies exist between laboratory findings (e.g., cytotoxicity, monomer release) and clinical outcomes (e.g., sensitivity), limiting direct clinical translation.

Moreover, contemporary luting agents encompass multiple categories, including conventional resin cements, self-adhesive resin cements, glass ionomer cements (GICs), and resin-modified glass ionomer cements (RMGICs), each with distinct chemical compositions and biological profiles. Despite this diversity, there is a lack of integrated synthesis evaluating their combined biological, chemical, and clinical effects on vital pulp.

Therefore, the present systematic review aims to address this gap by synthesizing evidence across different study designs.

Primary research question:

What is the effect of luting agents on the pulp of vital teeth?

Secondary questions:

How do luting agents affect pulpal cells in vitro and ex vivo models?

What is the histological pulpal response in in vivo studies?

What is the clinical impact in terms of postoperative sensitivity?

## 2. Materials and Methods

### 2.1. Protocol and Registration

This systematic review was developed using the Preferred Reporting Items for Systematic Reviews and Meta-Analyses (PRISMA) checklist [4]. An electronic search was conducted, and a thorough search strategy was developed using a modified PICO approach- the PIO criteria [5]. A comparator (C) was not considered mandatory because many of the eligible in vitro, ex vivo, animal, and observational studies evaluated the biological effects of a single luting material without the inclusion of a control or comparison group. Therefore, the review was designed to comprehensively synthesize evidence regarding the pulpal effects of luting agents across different study designs rather than to compare the efficacy or safety of specific interventions. The following is the PIO question: To what extent does the use of contemporary luting cements affect the pulp of vital teeth? (Table 1). The review question was intentionally designed to broadly evaluate the biological impact of luting agents on pulpal tissues rather than to compare the efficacy or safety of specific materials. A comprehensive systematic review including multiple study designs was therefore considered the most appropriate methodological approach, as it enabled the integration of mechanistic, histological, and clinical evidence and allowed a more holistic understanding of pulpal responses to luting agents across different experimental and clinical contexts.

This review was registered at the PROSPERO database under the number CRD420251156047 and can be accessed through the link: https://www.crd.york.ac.uk/PROSPERO/view/CRD420251156047 (accessed on 29 May 2026).

The review protocol was prospectively registered in the International Prospective Register of Systematic Reviews (PROSPERO) [6] under the identifier CRD420251156047.

### 2.2. Eligibility Criteria

Studies were selected according to predefined inclusion and exclusion criteria.

Inclusion criteria:

- In vitro, ex vivo, animal, human in vivo, observational, or randomized clinical studies;
- Studies evaluating the effects of luting agents on the pulp of vital teeth;
- Studies assessing biological, histological, biochemical, thermal, or clinical pulpal outcomes;
- Studies published irrespective of language;
- Studies published from 2000.

Exclusion criteria:

- Studies involving non-vital teeth or endodontically treated teeth;
- Case reports, case series, narrative reviews, systematic reviews, editorials, conference abstracts, letters, and expert opinions;
- Studies evaluating restorative materials other than luting agents without separate analysis of luting materials;
- Studies lacking pulp-related outcomes;
- Studies published before 2000.

### 2.3. Information Sources

This systematic review was conducted by searching the electronic databases PubMed (MEDLINE), the Cochrane Library, ScienceDirect, Web of Science, and Scopus, as well as the database ClinicalTrials.gov for Gray Literature. Additionally, the reference lists of included studies were manually screened. Moreover, the relevant results of previous research on the topic were included in the synthesis.

### 2.4. Search Strategy

Search strategies for each database were developed using Boolean operators, truncation, controlled terms (MeSH), and free-text keywords. No language restrictions were applied, but the date range was limited to 1 January 2000–31 March 2026, to assess contemporary materials. The search strategy was initially developed for PubMed using MeSH terms and free-text keywords: (luting cement*[Title/Abstract] OR “Dental Cements”[Mesh] OR “Cementation”[Mesh]) AND (“pulp vitality”[Title/Abstract] OR “pulp response”[Title/Abstract] OR “Dental Pulp”[Mesh]). The syntax was then adapted for each database (ScienceDirect, Scopus, Web of Science, Cochrane Library) to match their indexing systems and field tags. The full search strategies are available in Table 2.

### 2.5. Selection Process

Two reviewers (G.A. and M.D.) independently screened titles and abstracts. Full texts of potentially eligible studies were retrieved and assessed independently based on the eligibility criteria. Disagreements were resolved through discussion or consultation with a third reviewer (N.P.K.). No automated tools were used.

### 2.6. Data Collection Process

Two reviewers independently collected data using a pre-tested extraction form. The extracted data were verified for accuracy. Authors of the included studies were not contacted for clarification. No automated tools were used for data collection.

### 2.7. Data Items

Each study was extracted into a standardized form, including:

- Title, author, year, design/model (clinical trials/human in vivo/animal in vivo/in vitro/ex vivo)
- Luting agents/cement systems (names and categories when reported)
- Polymerization/cure protocol (light/dual/self; curing unit/irradiation strategy when relevant)
- Primary pulp-relevant outcomes and measurement methods

The outcomes of interest included: cytotoxicity, measured by cell viability, apoptosis, intrapulpal temperature rise and oxidative stress (in vitro studies); monomer diffusion through dentin; pulpal inflammation (in the ex vivo/histology studies); and postoperative sensitivity (in clinical trials). All available outcomes for each study were collected.

- Timepoints/follow-up duration
- Reporting items relevant to risk of bias

Other variables for which data were sought included: composition and monomer content, dentin pretreatment protocol, remaining dentin thickness, and funding sources.

Missing methodological details were recorded as “not reported.”

### 2.8. Study Risk of Bias Assessment

In vitro/Ex vivo studies: assessed with the QUIN tool [7].

Clinical trials: assessed with the Cochrane RoB 2 tool [8].

In vivo animal studies: assessed with the SYRCLE tool [9].

Νon-randomized human in vivo studies: assessed with the ROBINS-I [10] tool.

All studies were assessed by two independent reviewers (G.A. and M.D.), and disagreements were resolved by discussion.

### 2.9. Effect Measures

As the outcomes were heterogeneous (cell viability percentages, qualitative pulp histology, intrapulpal temperature rise, subjective sensitivity scores), no unified effect measure could be applied. Extracted outcomes were reported as summary statistics provided in the original studies.

### 2.10. Synthesis Methods

Eligibility for synthesis: Studies were grouped according to cement type (conventional vs. resin-based) and study design (in vitro, ex vivo, clinical).

Data preparation: No data transformation was required.

Presentation: Results were tabulated and described narratively; risk-of-bias tables were constructed.

Synthesis approaches: Meta-analysis was not feasible due to substantial methodological heterogeneity; therefore, narrative synthesis was performed.

Heterogeneity exploration: Differences in monomer type, dentin pretreatment, polymerization mode, and study model were examined qualitatively.

Sensitivity analyses: Not applicable because no pooled analyses were conducted.

### 2.11. Reporting Bias Assessment

Reporting bias was assessed qualitatively by comparing reported outcomes with those listed in the study methods sections. No formal statistical tests were possible. The methodological quality of the included studies was assessed according to study design. For each study design, the relevant domains and criteria for overall risk-of-bias judgments were predefined and applied consistently across all included studies.

### 2.12. Certainty Assessment

GRADE [11] was applied to a small set of critical outcomes: postoperative sensitivity in clinical trials, histologic pulpal inflammation in ex vivo and in vivo human/animal studies, intrapulpal temperature rise, and pulp cell cytotoxicity/oxidative stress markers in in vitro studies, with indirect clinical relevance.

Although GRADE was originally developed for evaluating patient-centered clinical outcomes, it was applied in the present review to provide a structured and transparent framework for assessing the certainty of the available evidence across all included studies. The assessment considered the standard GRADE domains (risk of bias, inconsistency, indirectness, imprecision, and publication bias), while acknowledging the predominantly laboratory-based nature of the evaluated outcomes. The studies assessed for certainty of evidence started with a baseline rating of HIGH for Randomized Clinical Trials (RCTs) and LOW for non-RCTs. A starting rating of ‘high quality’ of evidence was downgraded by one level for serious concerns (or by two levels for very serious concerns).

The assessment was performed by the same two independent reviewers (G.A. and M.D.).

## 3. Results

### 3.1. Study Selection

**Figure 1:**
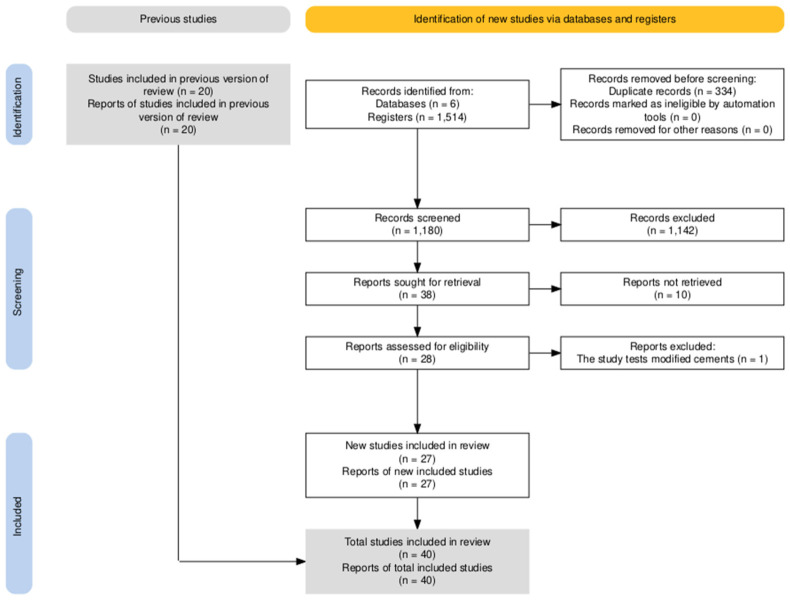
PRISMA 2020 flow diagram of the systematic review process.

Research of the electronic databases identified 1514 registers. Of these, 334 were removed as duplicates. Therefore, 1180 studies were screened based on Title and Abstract, as mentioned above, resulting in 38 studies. Unfortunately, 10 of them could not be retrieved as full-text articles, leaving 28 studies to be assessed for eligibility. After full-text screening, one study was excluded because it tested a modification to the cement’s composition, not the luting cement itself. Lastly, a literature search previously conducted by the authors for a systematic review on the same topic, using the keywords “luting cements,” “luting materials,” “luting agents,” “pulp,” “pulp response,” and “pulp vitality,” identified 20 studies that met the predefined eligibility criteria for inclusion. These studies formed the basis of the substantially revised and updated systematic review presented here. Among the two searches, seven studies were duplicates; therefore, the number of studies included in this review was 40. The PRISMA flowchart for the search results and study selection process is shown in Figure 1.

The types of these studies are diverse: 26 are in vitro, three are ex vivo, five are clinical trials, three are in vivo animal studies, and three are in vivo non-randomized human studies.

### 3.2. Overview of the Included Studies 

The included studies evaluated a wide range of luting agents, including resin-based cements, self-adhesive systems, glass ionomer cements (GICs), resin-modified GICs, and zinc phosphate cements.

Outcomes were grouped into: cytotoxicity and cellular response for the in vitro models, monomer diffusion and histological changes for ex vivo/in vivo studies, and postoperative sensitivity for clinical studies. A high degree of heterogeneity was observed in materials, protocols, and outcome measures. A summarizing table for the characteristics of included studies is shown in Table 3, while a detailed extraction table including sample size, model, cement brand, comparator, restoration type, follow-up, outcome methods, and main numerical findings is provided in Supplementary Table S1 in the Supplementary Materials.

### 3.3. Systematic Review of In Vitro and Ex Vivo Laboratory Studies

Twenty-nine included studies used in vitro or ex vivo models to investigate the biological, chemical, and thermal effects of luting agents on pulp-relevant tissues. These studies assessed cell viability, oxidative stress, inflammatory mediator expression, monomer diffusion, degree of conversion, dentin-barrier effects, microleakage, and intrapulpal temperature rise.

#### 3.3.1. Cytotoxicity and Pulp Cell Response

Most in vitro studies reported that resin-based luting agents produced material-dependent cytotoxic effects on pulp-derived or odontoblast-like cells. Reduced cell viability was observed after exposure to conventional and self-adhesive resin cements, with toxicity influenced by cement composition, curing protocol, and exposure time. This pattern was reported across studies using MDPC-23 odontoblast-like cells, human dental pulp cells, bovine pulp-derived cells, and dental pulp stem cells [12,15,16,18,19,20,23,24,25,36,38,47].

Several studies have linked cytotoxicity to resin monomers and incomplete polymerization. Bis-glycidyl methacrylate Bis-GMA exposure was associated with oxidative stress, cell-cycle alterations, and apoptosis in dental pulp cells, whereas Hydroxyethyl methacrylate (HEMA) and related monomers were implicated in matrix metalloproteinase expression and inflammatory pathways [28,29,34].

Glass ionomer and resin-modified glass ionomer materials generally showed variable but often lower cytotoxicity than resin cements, although cytotoxicity depended on formulation, fluoride release, and setting chemistry [17,22,37].

Overall, the laboratory evidence consistently demonstrated that resin-based luting cements exhibit greater cytotoxic potential than glass-ionomer-based materials. Across studies, cytotoxicity was influenced primarily by resin monomer composition, degree of conversion, and exposure duration. Studies employing dentin barrier models generally reported lower toxicity than direct-contact models, highlighting the protective role of dentin. Despite methodological heterogeneity, a consistent pattern emerged indicating that incomplete polymerization and higher concentrations of residual monomers were associated with increased adverse cellular responses.

#### 3.3.2. Influence of Dentin Barrier and Remaining Dentin Thickness

Studies utilizing a dentin barrier or transdentinal models demonstrated that dentin thickness plays a protective role. In particular, increasing the dentin thickness reduced cytotoxicity and limited diffusion of possible toxic components toward pulp cells [13,16,21,35]. The smear layer also appeared to have relevance. de Mendonça et al. reported that smear-layer thickness influenced transdentinal cytotoxicity, suggesting that dentin surface condition can modify pulpal exposure to cement-derived components [15].

Collectively, studies evaluating dentin-mediated effects consistently showed that increasing remaining dentin thickness attenuated cytotoxicity and reduced transdentinal diffusion of cement-derived substances. These findings were observed across different experimental models and cement categories, suggesting that dentin thickness is a key biological determinant of pulpal protection regardless of material type.

#### 3.3.3. Monomer Diffusion, Degree of Conversion, and Polymerization

Ex vivo diffusion studies confirmed that resin cement components can pass through dentin. Kerezoudi et al. used an artificial pulp chamber (APC) model to evaluate monomer leaching from resin cement, showing that monomers may diffuse toward the pulpal side depending on dentin pretreatment and time [3].

Degree of conversion was a recurring determinant of biological behavior. Nocca et al. showed that indirect restorative barriers affected degree of conversion, monomer release, and cytotoxicity of dual-cure resin cements [26]. Similarly, D’Alpino et al. reported activation-protocol-dependent cytotoxic effects of self-adhesive resin cements on odontoblastic cells [24].

More recent studies, including Josic et al. and Moon et al., further supported that polymerization strategy and cement category influence cytotoxicity, inflammatory response, and material biocompatibility [27,30].

Across studies, polymerization efficacy emerged as a major determinant of biological behavior. Studies consistently demonstrated that lower degrees of conversion were associated with increased monomer release, greater cytotoxicity, and enhanced inflammatory responses. Although differences existed among cement formulations and curing protocols, the overall evidence suggests that optimized polymerization protocols improve the biocompatibility of resin-based luting agents.

#### 3.3.4. Thermal Effects During Cementation

Three ex vivo studies evaluated intrapulpal temperature rise during luting or light curing. Taher et al. and Onisor et al. showed that light-curing protocols can increase intrapulpal temperature, and this is influenced by the light-curing unit, exposure time, cement type, and cooling conditions [32,33].

Kincses et al. demonstrated that both ceramic and dentin thickness, as well as resin-based luting agent type, affected intrapulpal temperature changes during luting of ceramic inlays [31]. These findings indicate that thermal risk is not determined by the cement alone but by the combined restorative-luting-curing system.

The available evidence consistently indicated that intrapulpal temperature rise is a multifactorial phenomenon influenced by curing protocols, restorative material thickness, dentin thickness, and cement composition. No study reported temperature increases sufficient to consistently predict irreversible pulpal damage, although prolonged light exposure and reduced dentin thickness appeared to increase thermal risk.

#### 3.3.5. Microleakage and Indirect Pulpal Risk

Gerdolle et al. evaluated microleakage of indirect composite inlays cemented with different luting agents. Although microleakage is not a direct measure of pulpal inflammation, it is clinically relevant because marginal leakage may facilitate bacterial penetration and secondary pulpal irritation [14].

### 3.4. Systematic Review of Animal Studies

Three included studies used animal in vivo models to evaluate pulpal tissue responses after cementation with resin-based luting agents [39,40,41]. Animal evidence suggests that these luting materials can induce mild to moderate pulpal response, but severe irreversible pulpal injury was not a consistent finding. The response appears to depend on material type, cementation protocol, sealing quality, bacterial penetration, and remaining dentin protection.

### 3.5. Systematic Review of Human In Vivo Studies

Collectively, human in vivo studies suggest that luting agents can induce measurable biological responses in the pulp, including mild inflammation and activation of inflammatory mediators. However, these responses are generally limited in magnitude, reversible over time, and dependent on dentin thickness and operative technique. No consistent evidence of irreversible pulpal damage attributable solely to luting agents was identified [1,2,46].

#### 3.5.1. Histological Pulpal Response

de Souza Costa et al. investigated the pulp response to resin-based luting systems in human teeth prepared for indirect restorations [1]. Histological analysis demonstrated that initial pulpal inflammation was generally mild and localized, with a tendency to decrease over time. Importantly, the degree of inflammation was strongly influenced by remaining dentin thickness (RDT), with deeper preparations showing more pronounced responses.

Similarly, Vigolo et al. evaluated pulpal response following cementation procedures and reported that, under clinically controlled conditions, luting agents did not induce significant long-term pulpal damage [46]. The findings suggested that pulpal tissues are capable of maintaining vitality when restorative procedures are performed appropriately, particularly when dentin protection is preserved.

#### 3.5.2. Biochemical and Neurogenic Response

Caviedes-Bucheli et al. assessed the expression of substance P, a neuropeptide associated with inflammation and pain, in pulpal tissues following exposure to different luting materials [2]. The study demonstrated increased substance P expression after cementation, indicating activation of neurogenic inflammatory pathways.

Taken together, human in vivo studies suggest that biological responses induced by luting agents are generally mild, transient, and strongly modulated by remaining dentin thickness and operative procedures. Despite differences in outcome measures, all studies indicated preservation of pulp vitality and absence of clinically significant irreversible damage.

### 3.6. Systematic Review of Clinical Trials

#### 3.6.1. Post-Operative Sensitivity

Clinical evidence on post-operative sensitivity was heterogeneous. Sensat et al. evaluated dentinal cold sensitivity following cementation with adhesive composite cements and reported that sensitivity outcomes differed between cement systems during early follow-up periods [45]. This suggests that luting materials and adhesive protocols may influence short-term pulpal symptoms. Similarly, Saad et al. reported variation between material groups [44]. These findings indicate that cement composition and clinical protocol may contribute to early sensitivity, particularly shortly after cementation.

In contrast, Kozmacs et al. reported long-term results of a prospective randomized split-mouth clinical study comparing crowns cemented with two luting agents [43]. The study found that hypersensitivity outcomes were generally comparable between groups over extended follow-up, suggesting that early sensitivity does not necessarily translate into persistent pulpal symptoms.

#### 3.6.2. Long-Term Clinical Outcomes and Pulp-Related Events

Jokstad et al. investigated long-term clinical performance of crowns cemented with glass-ionomer-based and conventional luting systems [42]. Although clinical failure and restoration-related outcomes were the primary focus, the absence of consistent long-term pulpal complications supports the view that appropriately used luting agents are generally compatible with maintenance of pulp vitality.

Behr et al. compared self-adhesive resin cement with zinc phosphate luting material in a prospective clinical trial [48]. The findings suggest no clear evidence that the resin cement produced worse pulp-related clinical outcomes than zinc phosphate under the tested conditions.

#### 3.6.3. Overall Synthesis of Clinical Evidence

Overall, clinical trials suggest that postoperative sensitivity after cementation may occur, particularly during early follow-up, but it is generally transient and material/protocol dependent. The evidence does not consistently show persistent pulpal symptoms or long-term pulp-related complications attributable solely to luting agents. Across randomized clinical trials, no particular cement category consistently demonstrated superior or inferior long-term pulp-related outcomes. These findings suggest that clinical technique, adhesive protocol, and preservation of dentin may have greater influence on pulpal health than cement composition alone.

The clinical findings also highlight an important contrast with laboratory studies: materials that show cytotoxic potential in vitro do not necessarily cause clinically relevant pulpal damage in vivo. This discrepancy may be explained by remaining dentin thickness, fluid movement, pulpal defense mechanisms, material handling, and sealing quality.

### 3.7. Results of Syntheses

Across all study designs, three major themes emerged: (1) resin-based materials exhibited greater biological activity than conventional cements; (2) remaining dentin thickness consistently modulated pulpal response; and (3) laboratory evidence demonstrated greater biological effects than were observed clinically.

There was considerable heterogeneity in experimental conditions, cement categories, dentin pretreatment, polymerization modes, and outcomes. In vitro studies consistently linked cytotoxicity to specific monomers and dentin permeability; clinical trials emphasized postoperative sensitivity.

No meta-analysis was performed because of heterogeneity in study designs and outcome metrics. The review, therefore, uses a narrative synthesis. Factors contributing to differences among study findings included: remaining dentin thickness, monomer composition, use of dentin pretreatment (acid etching vs. smear layer preservation), polymerization type (light-cure, chemical, dual-cure), and study type.

Sensitivity analysis was not applicable as no pooled quantitative data were synthesized.

### 3.8. Reporting Biases

In general, no direct evidence of selective reporting was identified. However, in vitro/ex vivo studies frequently lacked extractable statements on randomization of specimens and blinding, leading to overall ratings commonly driven by reporting limitations. Animal studies frequently lacked extractable details on allocation concealment, random housing, and blinding, resulting in overall “Unclear” patterns. Lastly, clinical/human studies included a mixture of randomized and non-randomized designs; across randomized studies, allocation concealment and protocol registration were rarely extractable, producing “Some concerns” patterns.

#### 3.8.1. Risk of Bias Assessment for In Vitro/Ex Vivo Studies

The current review provides a comprehensive assessment of the methodological quality of in vitro and ex vivo studies by applying a modified version of the QUIN tool, adapted for dental materials research [7]. Although assessments were performed at the individual study level, no meaningful variation in domain-level judgments was observed, supporting grouped presentation. A summary is provided in Table 4.

Most in vitro and ex vivo studies were judged to have high risk of bias, mainly because sample size calculation, randomization of specimens, and blinding of outcome assessment were rarely reported. Nevertheless, most studies clearly stated their aims, described experimental procedures, reported outcomes, and applied statistical analyses. Therefore, the laboratory evidence is useful for identifying mechanisms of pulpal irritation but remains indirect and should be interpreted cautiously when translated to clinical outcomes.

#### 3.8.2. Risk of Bias Assessment for Animal Studies

To comprehensively assess the methodological rigor of the 3 animal studies included in this review, the SYRCLE Risk of Bias tool (Systematic Review Centre for Laboratory Animal Experimentation) was applied [9]. A summary is provided in Table 5.

The animal studies were judged to have unclear to high risk of bias using the SYRCLE tool. The main limitations were insufficient reporting of random sequence generation, allocation concealment, random housing, and blinding of operators or outcome assessors. Although outcomes were generally reported, the lack of methodological transparency reduces confidence in the strength of conclusions drawn from these studies.

Therefore, animal evidence provides useful biological support for possible pulpal reactions to luting agents, but it should be considered supportive rather than definitive evidence.

#### 3.8.3. Risk of Bias Assessment for Human In Vivo Studies

This review provides a comprehensive assessment of the methodological quality of the 3 human in vivo studies by applying the ROBINS-I tool (Risk Of Bias In Non-randomized Studies of Interventions) [10]. A summary is provided in Table 6.

The human in vivo studies were assessed as having moderate risk of bias, primarily due to absence of randomization, potential confounding factors (e.g., operative variability), lack of blinding of outcome assessment, and limited reporting of methodological details.

Despite these limitations, these studies provide valuable direct evidence of pulpal responses under clinical conditions, although the strength of conclusions remains limited.

#### 3.8.4. Risk of Bias Assessment for Clinical Trials

The methodological quality of the five included randomized controlled trials was evaluated using the Cochrane RoB 2 tool [8]. A summary is provided in Table 7.

Clinical trials were generally judged as having some concerns using the RoB 2 tool. The main limitations were incomplete reporting of randomization procedures, unclear allocation concealment, lack of blinding, and absence of protocol registration. Missing outcome data were generally less concerning, especially in longer-term studies where follow-up was reported.

Therefore, clinical trial evidence provides the most directly relevant information for patient outcomes, but the certainty of conclusions remains limited by methodological reporting and heterogeneity in materials, follow-up periods, and outcome definitions.

### 3.9. Certainty of Evidence

The certainty of evidence was assessed for five critical outcomes using a GRADE-based approach: postoperative sensitivity, histological pulpal inflammation, monomer diffusion, cytotoxicity/cell viability, and intrapulpal temperature rise [11]. The overall certainty of evidence ranged from moderate to very low (Table 8).

The GRADE assessment showed that certainty of evidence ranged from moderate to very low. Postoperative sensitivity had moderate certainty, as it was supported by five clinical trials and represented a direct patient-relevant outcome; however, it was downgraded for risk of bias due to limitations in randomization, allocation concealment, and blinding.

Histological pulpal inflammation, monomer diffusion, cytotoxicity/cell viability, and intrapulpal temperature rise were all rated as very low certainty. These outcomes were downgraded mainly due to risk of bias, indirectness, and imprecision. Cytotoxicity/cell viability was additionally downgraded for inconsistency because of variation in cell models, materials, exposure protocols, and outcome measures.

Overall, clinical evidence for postoperative sensitivity provides the strongest basis for interpretation, whereas laboratory outcomes should be considered supportive biological evidence rather than direct proof of clinical pulpal damage.

## 4. Discussion

The present systematic review synthesized evidence from 40 studies assessing the effects of luting agents on the pulps of vital teeth across experimental and clinical settings. The included studies encompassed a broad range of experimental and clinical designs, including in vitro, ex vivo, animal, observational human studies, and randomized clinical trials. Given this methodological heterogeneity, it is important to consider the hierarchy of evidence when interpreting the findings. Randomized clinical trials provide the highest level of clinical evidence, followed by observational human studies, animal experiments, ex vivo investigations, and in vitro studies. This hierarchy should be taken into consideration when interpreting the overall results, as the strength and clinical relevance of the evidence vary substantially across study designs.

### 4.1. Interpretation of Laboratory Evidence

Overall, in vitro and ex vivo evidence suggests that luting agents may affect pulp-related tissues through three main mechanisms: chemical toxicity from residual monomers, biological activation of oxidative or inflammatory pathways, and thermal stress during polymerization. Resin-based cements showed the greatest variability, with effects strongly dependent on composition, activation mode, degree of conversion, and dentin thickness. Glass ionomer-based materials generally appeared more favorable biologically, but their effects were not uniform across studies.

### 4.2. Translation to In Vivo and Clinical Conditions

Evidence from animal studies suggests that luting agents can induce mild to moderate pulpal responses, but severe irreversible pulpal injury was not a consistent finding. The response appears to depend on material type, cementation protocol, sealing quality, bacterial penetration, and remaining dentin protection.

However, animal studies should be interpreted with caution because their findings are not directly comparable to human clinical outcomes. Differences in tooth anatomy, pulp physiology, restorative procedures, and follow-up conditions limit direct translation.

Similarly, human in vivo studies suggest that luting agents can induce measurable biological responses in the pulp, including mild inflammation and activation of inflammatory mediators. However, these responses are generally limited and reversible over time.

These findings provide an important bridge between laboratory and clinical evidence, supporting the concept that while luting agents may have biological effects, the clinical impact is moderated by physiological and procedural factors.

Clinical trials provided the most directly relevant evidence. Postoperative sensitivity was reported in several studies, particularly during early follow-up [44,45]. However, long-term outcomes were generally favorable, with no consistent evidence of persistent pulpal symptoms or increased risk of pulpal pathology [42,43,48].

### 4.3. Biological Meaning of Outcomes

A critical consideration when interpreting the evidence is the difference in biological meaning across study outcomes. In vitro measures, such as cell viability, oxidative stress, or inflammatory marker expression, reflect cellular responses under controlled conditions and do not equate to clinical endpoints, such as pulp vitality or pain.

Therefore, reduced cell viability in laboratory models should not be interpreted as direct evidence of pulpal necrosis or irreversible damage. Instead, these findings should be viewed as indicators of potential biological pathways that may contribute to pulpal irritation under certain conditions.

### 4.4. Role of Dentin Thickness and Clinical Technique

Across all study designs, remaining dentin thickness emerged as the most important determinant of pulpal response. Increased dentin thickness reduces monomer diffusion, attenuates cytotoxic effects, and limits thermal impact [1,3,13].

However, its influence should be interpreted within the broader context of material-related and procedural variables. Remaining dentin thickness primarily functions as a biological barrier by limiting the transdentinal diffusion of potentially cytotoxic components and attenuating thermal transmission to the pulp. Nevertheless, the composition of luting agents, particularly the nature and concentration of resin monomers, substantially influences their biological profile and capacity to induce cytotoxic, oxidative, or inflammatory responses. In addition, polymerization protocols are a critical procedural factor, as the degree of conversion directly affects the amount of residual unpolymerized monomers available to diffuse through dentin. Inadequate polymerization may therefore increase pulpal exposure to biologically active substances and potentially induce adverse cellular responses. Collectively, these findings suggest that pulpal outcomes following cementation procedures are determined by a complex interaction among substrate-related factors (remaining dentin thickness), material-related characteristics (cement composition), and technique-sensitive variables, including polymerization strategy and clinical application protocols.

### 4.5. Heterogeneity of Evidence

A major limitation of the available literature is the substantial heterogeneity across studies. Differences in study design (in vitro, ex vivo, animal, clinical), luting material composition, polymerization protocols, dentin pretreatment strategies, and outcome measures limit direct comparison and preclude quantitative synthesis in this review.

This heterogeneity also explains the variability in reported outcomes, particularly in clinical studies evaluating postoperative sensitivity.

### 4.6. Risk of Bias and Strength of Evidence

In vitro and ex vivo studies showed predominantly high risk of bias due to lack of randomization, blinding, and sample size calculation, although reporting of outcomes and statistical analysis was generally adequate. Animal studies demonstrated unclear to high risk of bias, primarily due to insufficient reporting of allocation procedures and blinding.

Human in vivo studies were assessed as having a moderate risk of bias, mainly due to confounding factors such as cavity depth, dentin thickness, and operator variability. Clinical trials were generally judged as having “some concerns,” reflecting limitations in reporting of randomization and blinding.

The overall certainty of evidence ranged from very low to moderate; therefore, conclusions should be interpreted with caution. Postoperative sensitivity showed the highest certainty of evidence, rated as moderate, because it was supported by direct clinical evidence from the five clinical trials. However, histological pulpal inflammation, monomer diffusion, cytotoxicity/cell viability, and intrapulpal temperature rise were rated as very low certainty, mainly due to risk of bias, indirectness, and imprecision. Therefore, laboratory outcomes should be interpreted as supportive biological evidence rather than direct proof of clinical pulpal damage. This highlights the need for better-designed clinical studies with standardized outcomes, clear reporting of dentin thickness, and longer follow-up.

### 4.7. Limitations of the Review

This systematic review has several limitations. First, the inclusion of different study designs introduces indirectness, as laboratory and animal models do not fully replicate clinical conditions. Second, heterogeneity in study protocols and outcomes prevented meta-analysis. Third, incomplete reporting in several studies may have influenced the risk-of-bias evaluation. Finally, another limitation of this review is the application of the GRADE framework to predominantly laboratory-derived evidence. Because GRADE was developed primarily for patient-centered clinical outcomes, some of its domains are less readily applicable to preclinical studies. In particular, the assessment of indirectness is challenging, as laboratory outcomes represent surrogate rather than clinical endpoints, while the evaluation of publication bias is limited by the absence of trial registration and the underreporting of negative laboratory findings. Therefore, although GRADE provided a structured and transparent framework for assessing the certainty of the available evidence, the resulting certainty ratings should be interpreted with caution.

### 4.8. Clinical Implications

From a clinical perspective, the findings suggest that luting agents are generally safe when used under appropriate conditions. The choice of cement should be guided by the clinical situation, with particular attention to dentin thickness and proper application protocols.

Dual-cure and self-adhesive resin cements seem to provide advantageous biocompatibility profiles; nevertheless, their biological behavior is affected by polymerization conditions and material composition. It is imperative to ensure sufficient dentin protection and to employ appropriate curing techniques to mitigate pulpal irritation.

### 4.9. Future Research

Future research should focus on minimizing heterogeneity and enhancing methodological rigor. Well-structured clinical trials utilizing standardized protocols, extended follow-up periods, and explicit reporting of pulpal outcomes are essential. Furthermore, studies that incorporate biological markers alongside clinical outcomes may significantly advance understanding of the correlation between laboratory findings and patient-reported symptoms.

## 5. Conclusions

Luting agents may elicit biological responses in the pulp of vital teeth; however, these effects are generally mild, transient, and highly dependent on clinical conditions. While in vitro and ex vivo studies demonstrate cytotoxicity, monomer diffusion, and thermal effects, in vivo and clinical evidence indicate that pulpal responses are typically reversible and rarely result in long-term complications.

Remaining dentin thickness appears to be the most critical factor in modulating pulpal response, while material composition and polymerization protocols also influence outcomes. Among contemporary materials, dual-cure and self-adhesive resin cements show favorable biocompatibility when properly applied.

However, given that a substantial proportion of the available evidence is derived from in vitro and ex vivo studies, and considering the overall low certainty of the evidence, these findings should be interpreted with caution. Careful clinical technique and preservation of dentin remain essential. Further well-designed clinical studies are needed to strengthen the evidence base and support more definitive conclusions regarding material selection.

## Figures and Tables

**Table 1:** Search strategy according to the PIO criteria.

PIO Component	Description
Population	Vital teeth, Pulp-relevant in vitro/ex vivo models
Intervention	Use of contemporary luting materials (resin cements, conventional and resin-modified glass ionomer cements)
Outcome	Cell cytotoxicity, pulp inflammation, postoperative sensitivity

**Table 2:** Electronic search strategy and search dates for each database.

Database	Search Date	Search Strategy
PubMed	1 January 2000–31 March 2026	(luting cement*[Title/Abstract] OR “Dental Cements”[Mesh] OR “Cementation”[Mesh]) AND (“pulp vitality”[Title/Abstract] OR “pulp response”[Title/Abstract] OR “Dental Pulp”[Mesh])
Scopus	1 January 2000–31 March 2026	(“luting cement” OR “dental cements” OR “cementation”) AND (“pulp vitality” OR “pulp response” OR “dental pulp”)
Web of Science	1 January 2000–31 March 2026	(“luting cement” OR “dental cements” OR “cementation”) AND (“pulp vitality” OR “pulp response” OR “dental pulp”)
ScienceDirect	1 January 2000–31 March 2026	(“luting cement*” OR “dental cements” OR “cementation”) AND (“pulp vitality” OR “pulp response” OR “dental pulp”)
Cochrane Library	1 January 2000–31 March 2026	#1: “luting cement*”:ti,ab,kw #2: MeSH descriptor: [Dental Cements] explode all trees #3: MeSH descriptor: [Cementation] explode all trees #4: #1 OR #2 OR #3 #5: “pulp vitality”:ti,ab,kw #6: “pulp response”:ti,ab,kw #7: MeSH descriptor: [Dental Pulp] explode all trees #8: #5 OR #6 OR #7 #9: #4 AND #8
ClinicalTrials.gov	1 January 2000–31 March 2026	Condition or disease: Dental Pulp Other terms: luting cement OR dental cement OR cementation

Abbreviations: ti = title; ab = abstract; kw = keyword; MeSH = Medical Subject Headings. In the Cochrane Library strategy, #1–#9 denote sequential search sets combined using Boolean operators. The asterisk (*) denotes truncation (wildcard) to retrieve words with the same root (e.g., “cement” and “cements”).

**Table 3:** Characteristics of included studies.

Author (Year)	Study Design	Model	Luting Material(s)	RDT	Outcomes	Key Findings
Annunziata et al. (2006) [12]	In vitro	Cell culture	Resin cements	–	Cytotoxicity	Material-dependent toxicity
de Souza Costa et al. (2006) [1]	Human in vivo	Teeth	Resin cement	Yes	Histology	Mild inflammation, reversible
Galler et al. (2005) [13]	In vitro	Dentin barrier	Multiple	Yes	Cytotoxicity	RDT reduces toxicity
Gerdolle et al. (2005) [14]	In vitro	Cells	Resin cement	–	Cytotoxicity	Monomer-related toxicity
de Mendonça et al. (2012) [15]	In vitro	Cells	Resin cement	–	Cytotoxicity	Reduced viability
Garcia et al. (2015) [16]	In vitro	Cells	Resin cement	–	Cytotoxicity	Material-dependent response
Kanjevac et al. (2012) [17]	In vitro	Cells	Resin cement	–	Inflammation	Increased mediators
Kong et al. (2009) [18]	In vitro	Cells	Resin cement	–	Cytotoxicity	Polymerization-dependent toxicity
Schmid-Schwap et al. (2009) [19]	In vitro	Cells	Resin cement	–	Cytotoxicity	Dose-dependent effect
Ülker et al. (2012) [20]	In vitro	Cells	Resin cement	–	Cytotoxicity	Variable toxicity
Soares et al. (2015) [21]	In vitro	Cells	Resin cement	–	Cytotoxicity	Improved formulations
Ersahan et al. (2019) [22]	In vitro	Cells	GIC/RMGIC	–	Cytotoxicity	Lower toxicity vs. resin
Alvarez et al. (2019) [23]	In vitro	Cells	Resin cement	–	Cytotoxicity	Reduced viability
D’Alpino et al. (2017) [24]	In vitro	Cells	Resin cement	–	Cytotoxicity	Monomer effects
Kerezoudi et al. (2016) [3]	Ex vivo	Dentin disks	Resin cement	Yes	Diffusion	Monomer penetration
Nakagawa et al. (2015) [25]	In vitro	Cells	Adhesives	–	Cytotoxicity	Favorable biocompatibility
Nocca et al. (2015) [26]	In vitro	Cells	Resin cement	–	Diffusion	Barrier effect
Josic et al. (2024) [27]	In vitro	Cells	Resin cement	–	Inflammation	↑ IL-6 expression
Lee et al. (2017) [28]	In vitro	Cells	Resin cement	–	Cytotoxicity	Dose-dependent
Sun et al. (2017) [29]	In vitro	Cells	Resin cement	–	Cytotoxicity	Significant toxicity
Moon et al. (2026) [30]	In vitro	Cells	Resin cement	–	Cytotoxicity	Material-dependent
Kincses et al. (2023) [31]	In vitro	Cells	Resin cement	–	Cytotoxicity	Moderate toxicity
Onisor et al. (2011) [32]	Ex vivo	Teeth	Resin cement	Yes	Temperature	Intrapulpal rise
Taher et al. (2008) [33]	Ex vivo	Teeth	Resin cement	Yes	Temperature	Heat increase
Chang et al. (2010) [34]	In vitro	Cells	Bis-GMA	–	ROS/apoptosis	Oxidative stress
Hadjichristou et al. (2021) [35]	In vitro	Cells	Resin cement	–	Cytotoxicity	Material-dependent
Hiraishi et al. (2003) [36]	In vitro	Cells	Adhesives	–	Cytotoxicity	Toxic effects
Gonzalez et al. (2018) [37]	In vitro	Cells	Resin cement	–	Cytotoxicity	Reduced viability
Malkoc et al. (2015) [38]	In vitro	Cells	Resin cement	–	Cytotoxicity	Variable response
Uzzaman et al. (2005) [39]	Animal	Dogs	Resin cement	Yes	Histology	Mild inflammation
Bezzon et al. (2015) [40]	Animal	Animals	Resin cement	Yes	Histology	Reversible response
Shimada et al. (2007) [41]	Animal	Animals	Resin cement	Yes	Histology	Mild changes
Jokstad et al. (2004) [42]	RCT	Patients	RMGIC/Zn phosphate	–	Sensitivity	No long-term difference
Kozmacs et al. (2017) [43]	RCT	Patients	Resin/Zn phosphate	–	Sensitivity	Comparable outcomes
Saad et al. (2010) [44]	Clinical	Patients	Multiple	–	Sensitivity	Short-term increase
Sensat et al. (2002) [45]	RCT	Patients	Resin cement	–	Sensitivity	Early sensitivity
Vigolo et al. (2007) [46]	Human In vivo	Patients	Resin cement	Yes	Histology	No long-term damage
Caviedes-Bucheli et al. (2013) [2]	Human In vivo	Patients	GIC/resin	–	Substance P	↑ inflammation
de Mendonça et al. (2007) [47]	In vitro	Cells	Hard-setting cements, including resin-based materials	–	Cytotoxicity	Material-dependent/resin-containing materials showed greater adverse effects on odontoblast-like cells
Behr et al. (2009) [48]	RCT	Patients	Resin vs. conventional	–	Sensitivity	Comparable clinical outcomes

RDT: Remaining dentin thickness; RCT: Randomized clinical trial; GIC: Glass ionomer cement; RMGIC: Resin-modified glass ionomer cement.

**Table 4:** Summary of Risk of Bias assessment using the modified QUIN Tool.

Domain	Judgment	Justification
Aim clearly stated	Low	All studies clearly reported objectives and experimental purpose.
Sample size calculation	High	No study reported an a priori sample size or power calculation.
Randomization of specimens	High	No explicit random allocation of specimens was described.
Blinding of outcome assessment	High	No study reported blinding of evaluators.
Standardization of procedures	Moderate	Experimental protocols described; operator calibration not consistently reported.
Outcome reporting	Low	Outcomes and methods clearly defined and reported.
Statistical analysis	Low	Appropriate statistical tests were generally described.

**Table 5:** Summary of Risk of Bias assessment using the SYRCLE Tool.

Animal Study	D1	D2	D3	D4	D5	D6	D7	D8	D9	D10	Overall
Bezzon et al. 2015 [40]	NI	NI	NI	NI	High	NI	High	Low	Low	NI	High
Shimada et al. 2007 [41]	Low	NI	NI	NI	High	NI	High	Low	Low	NI	High
Uzzaman et al. 2005 [39]	NI	NI	NI	NI	High	NI	High	Low	Low	NI	High

NI: Insufficient Information; D1: Sequence Generation (Selection Bias); D2: Baseline Characteristics (Selection Bias); D3: Allocation Concealment (Selection Bias); D4: Random Housing (Performance Bias); D5: Blinding of Caregivers/Investigators (Performance Bias); D6: Random Outcome Assessment (Detection Bias); D7: Blinding of Outcome Assessment (Detection Bias); D8: Incomplete Outcome Data (Attrition Bias); D9: Selective Reporting (Reporting Bias); D10: Other Bias.

**Table 6:** Summary of Risk of Bias assessment using the ROBINS-I Tool.

Study	Bias Due to Confounding	Bias in Selection of Participants	Bias in Classification of Interventions	Bias Due to Deviations from Intended Interventions	Bias Due to Missing Data	Bias in Measurement of Outcomes	Bias in Selection of Reported Results	Overall Risk of Bias
de Souza Costa et al. (2006) [1]	Moderate	Moderate	Low	Moderate	Low	Moderate	Moderate	Moderate
Vigolo et al. (2007) [46]	Moderate	Moderate	Low	Moderate	Low	Moderate	Moderate	Moderate
Caviedes-Bucheli et al. (2013) [2]	Moderate	Moderate	Low	Moderate	Low	Moderate	Moderate	Moderate

**Table 7:** Summary of Risk of Bias assessment using the Cochrane RoB 2 Tool.

Study	Bias Arising from the Randomization Process	Bias Due to Deviations from Intended Interventions	Bias Due to Missing Outcome Data	Bias in Measurement of the Outcome	Bias in Selection of the Reported Result	Overall Risk of Bias
Sensat et al. (2002) [45]	Some concerns	Some concerns	Some concerns	Some concerns	Some concerns	Some concerns
Jokstad et al. (2004) [42]	Low	Low	Low	Low	Some concerns	Low
Behr et al. (2009) [48]	Some concerns	Some concerns	Low	Some concerns	Some concerns	Some concerns
Saad et al. (2010) [44]	Some concerns	Some concerns	Some concerns	Some concerns	Some concerns	Some concerns
Kozmacs et al. (2017) [43]	Some concerns	Some concerns	Low	Some concerns	Some concerns	Some concerns

**Table 8:** Summary of Findings and Certainty of Evidence Using the GRADE Approach.

Critical Outcome	Studies Contributing to the Outcome	Risk of Bias	Inconsistency	Indirectness	Imprecision	Publication Bias	Overall Certainty of Evidence
Postoperative sensitivity	5 clinical trials	Serious	Not Serious	Not serious	Not Serious	Not serious	Moderate
Histological pulpal inflammation	3 human/animal histology studies + 1 human biomarker study	Serious	Not serious	Serious	Serious	Not serious	Very low
Monomer diffusion	2 ex vivo/barrier-model studies + supportive laboratory evidence	Serious	Not serious	Serious	Serious	Not serious	Very low
Cytotoxicity/cell viability	Majority of in vitro/ex vivo studies	Serious	Serious	Serious	Serious	Not serious	Very low
Intrapulpal temperature rise	3 ex vivo studies	Serious	Not serious	Serious	Serious	Not serious	Very low

## Data Availability

Upon request to the corresponding author, the data are available for use.

## Supplementary Materials

The following supporting information can be downloaded at: https://www.mdpi.com/article/10.3390/dj14070442/s1, Table S1. Characteristics of Included Studies, Table S2. PRISMA 2020 for Abstracts checklist, Table S3. PRISMA 2020 checklist.

## Abbreviations

The following abbreviations are used in this manuscript:

GIC	Glass-ionomer cement
RMGIC	Resin-modified glass-ionomer cement
RDT	Remaining dentin thickness
RCT	Randomized Clinical Trial
BIS-GMA	Bis-glycidyl methacrylate
HEMA	Hydroxyethyl methacrylate
