# Supplementary material for: The Impact of Luting Agents on the Pulp of Vital Teeth: A Systematic Review
Source: Dent J (Basel). 2026 Jul 15;14(7):442. doi: 10.3390/dj14070442 (PMC13408631; doi:10.3390/dj14070442)
Supplement: Supplementary file 1 [file dentistry-14-00442-s001.zip › dentistry-4377665-supplementary.pdf]

Supplementary Table S1. Characteristics of Included Studies

Author (Year)	Study design	Sample size	Tooth / cell model	Restoration type	Exact cement / material	Comparator	RDT / dentin condition	Polymerization / application protocol	Follow-up / exposure time	Outcome measurement method	Main numerical findings	Main conclusion	Risk-of-bias assessment tool	Overall risk-of-bias judgment
Annunziata et al. (2006) [20]	In vitro	Primary cultures; 10^4 cells/well	Human pulp and gingival fibroblasts	Composite inlays	Light-cured composite used as bonding system	Shielded vs unshielded composite polymerization	Composite inlay shielding	Photo-cured with/without 2 mm composite inlay	72 h	MTT assay	Higher cytotoxicity for inlay-shielded specimens	Light-shielding increased cytotoxicity	Modified QUIN	High risk
de Souza Costa et al. (2006) [1]	Human in vivo	34 sound human premolars	Human premolars from patients aged 12–18 years	Inlays	Variolink II + Excite	Two resin cement protocols	RDT-dependent	Manufacturer instructions	7 and 60 days	Histology	At 7 days, RelyX Unicem induced mild inflammation in 2 samples, whereas Variolink II induced moderate inflammation in 2 samples; pulpal response decreased at 60	Mild inflammation, reduced over time; influenced by RDT	ROBINS-I	Moderate risk

											days			
Galler et al. (2005) [30]	In vitro	Three-dimensional cultures with bovine dentin discs; exact number of discs not explicitly extractable from OCR text	3D pulp-derived cells + dentin discs	None	Syntac Classic; Prompt L-Pop; Vitrebond	Material groups	100–500 µm dentin	Applied on dentin discs	24 h	Dentin barrier test / cell survival assay	Cell survival increased as dentin thickness increased from 100 to 500 µm	Greater dentin thickness reduced toxicity	Modified QUIN	High risk
Gerdolle et al. (2005) [39]	In vitro	40 extracted human mandibular third molars	Human teeth	Indirect composite inlays	Variolink II; Panavia F; Fuji Plus	Four luting agents	Preparation overlapped CEJ	Standard cementation	Post-thermocycling	Microleakage	216 measurements per luting agent	Microleakage varied by luting agent	Modified QUIN	High risk
de Mendonça et al. (2012) [18]	In vitro / transdentinal	n=8 / n=10 / n=6 groups reported	MDPC-23 cells + dentin discs	None	Vitrebond	Smear-layer thickness groups	Dentin barrier present	Transdentinal application	24 h	MTT + bond strength	Forty teeth were used for bond-strength testing and 8 specimens for SDH/cell-metabolism	Smear layer influenced transdentinal cytotoxicity and bonding	Modified QUIN	High risk

											analysis		
Garcia et al. (2015) [14]	In vitro / transdentinal	n=10 per group	MDPC-23 and HDPCs	None	RelyX U200; RelyX ARC; RelyX Luting 2	Material groups	Artificial pulp chamber / dentin discs	According to manufacturers	24 h	Cell viability / morphology	RelyX Luting 2 showed lower transdentinal cytotoxicity than the resin-based luting cements in the tested model	Material-dependent transdentinal cytotoxicity	Modified QUIN	High risk
Kanjevac et al. (2012) [28]	In vitro	1×10^4 cells/100 µL	Human dental pulp stem cells	None	Vitrebond; Fuji Plus and other GICs	Material groups	Not applicable (direct cell model)	Eluate-based exposure	24 h	Cytotoxicity / fluoride correlation	Cytotoxicity correlated with fluoride release across the tested glass-ionomer materials	Cytotoxicity correlated with fluoride release	Modified QUIN	High risk
Kong et al. (2009) [15]	In vitro	10^4 cells/well	Human dental pulp cells	None	Panavia F 2.0 and comparator resin cements	Material groups	Not applicable (direct cell model)	Polymerized specimens	Eluates prepared for 72 h; cell exposure after 24 h attachment	Cell viability	At 100% eluate concentration, relative growth rate was 74.42% for Panavia F, 85.54% for Super-	Polymerization influenced toxicity	Modified QUIN	High risk

											Bond C&B, and 82.39% for Chemiace II			
Schmid-Schwap et al. (2009) [19]	In vitro	Sample size determined statistically	Cell culture	None	RelyX Unicem; MaxCem; Variolink II; Panavia F 2.0	Four cement categories	Not applicable (direct cell model)	Prepared per manufacturers	72 h	Cytotoxicity	Dose–response relationships were observed across four cement categories; exact values varied by concentration	Dose- and material-dependent cytotoxicity	Modified QUIN	High risk
Ülker et al. (2012) [16]	In vitro	Extract-based testing on human and bovine pulp-derived cells; exact total number not explicitly extractable from OCR text	Human and bovine pulp-derived cells	None	RelyX Unicem Clicker; MaxCem; Panavia F 2.0	Material groups	Not applicable (extract-based direct cell model)	Prepared in Teflon rings	24 h for cytotoxicity; 1 h for ROS generation	Cell reaction / cytotoxicity	Cytotoxicity ranking: RX < BII < PF < BC; 1 mmol/L TEGDMA approximately doubled ROS in bovine pulp-derived cells	Variable pulp-cell reaction across cements	Modified QUIN	High risk

Soares et al. (2015) [31]	In vitro / transdentinal	3 groups; MDPC-23 cells	Rat dental papilla-derived MDPC-23 cells + dentin discs	None	HEMA-free resin-based luting cements; RelyX Unicem comparator	Application protocol groups	Dentin discs	Different dentin-surface protocols	24 h	Cytotoxicity / cell morphology / SEM	50 dentin discs approximately 0.5 mm thick were used; SDH activity and ALP were influenced by dentin-surface protocol	Application protocol affected cytocompatibility	Modified QUIN	High risk
Ersahan et al. (2019) [27]	In vitro	Two cell systems tested in multiwell format; exact total number of wells not fully extractable from OCR text	Mouse fibroblasts and human dental pulp cells	None	Contemporary GICs / RMGICs	Material groups	Not applicable (direct cell culture model)	Material eluates	24 h, 48 h, and 72 h	MTT assay in mouse fibroblasts and human dental pulp cells	Toxicity varied between materials and between fibroblast and HDPC cultures across 24–72 h	Toxicity varied; generally lower than many resin cements	Modified QUIN	High risk
Alvarez et al. (2019) [12]	In vitro	10^4 cells/well	MDPC-23 odontoblast-like cells	None	MaxCem Elite; RelyX U200	Two self-adhesive cements	Not applicable (direct cell model)	Photoactivated per manufacturers	4 h exposure for oxidative-stress/gene-expression assays;	Oxidative stress, proliferation, alizarin red	MAX induced significantly higher oxidative stress than U200 and upregulated pro-	MAX induced higher oxidative stress than U200	Modified QUIN	High risk

									24 h incubation before mineralization-related assays		inflammatory cytokines such as TNF-α			
D'Alpino et al. (2017) [13]	In vitro	Five cements tested across three polymerization protocols	Odontoblast-like cells	None	Commercial self-adhesive cements including RelyX U200 and MaxCem Elite	Activation protocols	Not applicable (direct cell model)	Different activation modes	24 h specimen storage before testing	Annexin V / viability	All cements significantly reduced viability; CSA and U200 were least harmful; BSE, GCE, and MAX caused higher cell death, especially under chemical activation	Cytotoxicity depended on activation protocol	Modified QUIN	High risk
Kerezoudi et al. (2016) [3]	Ex vivo	10 dentin discs	Human dentin discs / APC	None	Multilink Automix	Pretreatment / time groups	Positive pulpal pressure model	Transdentinal diffusion model	9 time intervals up to 10 days	HPLC	HEMA detected; BPA/UDMA/Bis-GMA not detected	Monomer diffusion was monomer-specific	Modified QUIN	High risk
Nakagawa et al. (2015)	In vitro	42 specimens; 3×10^4	Cell culture / material interface	None	4-META/MMA-TBB	Comparator luting material	Not applicable (materi	Manufacturers' instructions	30 min to 24 h depending on	Cell attachment / biocompatibility	Degree of polymerization of 4-	Favorable biocompatibility	Modified QUIN	High risk

[21]		cells/cm²			resin	s	al–cell contact model)		assay		META/MMA-TBB resin was 82% at 30 min versus 66% for PMMA-MMA; comparator resins produced 10–20 times more free radicals			
Nocca et al. (2015) [33]	In vitro / barrier model	10^4 cells	Cell-based barrier model	Indirect restoration simulation	Cement-One; SmartCem2	Barrier thickness / material groups	2 and 4 mm barriers	Dual-cure through indirect materials	Immediate and eluate testing	Degree of conversion, monomer release, cytotoxicity	Not explicitly stated in extractable article text	Barriers affected conversion, release, and toxicity	Modified QUIN	High risk
Josic et al. (2024) [34]	In vitro	32 wells for each cement; 2 specimens per cement and polymerization protocol for some analyses; n = 16 for selected light-cured	Human dental pulp cells	None	Universal resin composite cements (Panavia-related category reported)	Material groups	Not applicable (direct cell model)	Prepared per manufacturers	12 h and 24 h; additional aging/biofilm time points	Cytotoxicity / cytokines / microbiology	Light-curing decreased biofilm formation by approximately 33%; cytotoxicity depended on polymerization protocol and observati	Polymerization and material influenced IL-6 and cytotoxicity	Modified QUIN	High risk

		groups									on time			
Lee et al. (2017) [26]	In vitro	Experiments performed in triplicate (n = 5 reported for some analyses)	DPSCs / monocytes	None	ZOE-based luting-relevant materials	Material groups	Not applicable (extract-based direct cell model)	ISO-based specimen preparation	24 h extract preparation and subsequent exposure assays	Cell viability / inflammatory response	Zn^{2+} and eugenol were detected; EC50 of Zn^{2+} was about 5–8 ppm; significant cytotoxicity was observed during the early setting stage	Dose-dependent biological effects	Modified QUIN	High risk
Sun et al. (2017) [25]	In vitro	10^6 cells / dish	Human pulp cells and odontoblast-like cells	None	HEMA	Exposure groups	Not applicable (direct cell model)	24 h exposure	MMP expression / molecular assays	Primary pulp-cell culture with molecular assays for MMP-2 and MMP-9 expression	Sixteen teeth from donors aged 12–25 years provided pulp cultures; HEMA altered MMP-related molecular pathways	HEMA exposure was associated with biologically relevant molecular changes in pulp-related cells, supporting a mechanistic role for resin monomers in pulpal irritation.	Modified QUIN	High risk

Moon et al. (2026) [35]	In vitro	Ten specimens per cement type for time-point testing; n = 4 for degree-of-conversion on image analysis	Eluate-based pulp-related model	None	ARC / SARC / RMGIC categories	Cement categories	Not applicable (eluate-based direct cell model)	Working/ setting-time based specimen prep	24 h, 48 h, and 2 weeks	GC/MS, flow cytometry	ARC and SARC showed marked cytotoxicity at low degree of conversion, whereas RMGIC showed minimal cytotoxicity across tested conversion levels	Biocompatibility depended on cement category and conversion	Modified QUIN	High risk
Kincses et al. (2023) [38]	In vitro	120 measurements	Extracted teeth, ceramic-dentin-cement model	Ceramic inlays	Resin-based luting agents	Ceramic thickness / dentin thickness / cement groups	Variable dentin thickness	Light-curing through ceramic	Immediate	Intrapulpal temperature	Approx. 5.4–7.9 °C rise	Temperature rise strongly depended on dentin and ceramic thickness	Modified QUIN	High risk
Onisor et al. (2011) [37]	Ex vivo	1 extracted human molar model	Extracted human molar	Ceramic onlay	Light-cured luting material system	Different curing regimens	Intact vs onlay-restored tooth	3×120 s irradiation ± cooling	Immediate	Intrapulpal temperature	Max 6.2 °C / 7.7 °C intact/restored	Thermal rise depended on irradiation and cooling	Modified QUIN	High risk
Taher et al. (2008) [36]	Ex vivo	60 specimens	Human dentin discs + composite discs	Indirect luting model	Variolink II; Choice	Different light-curing units	Human dentin discs	QTH, LED, PAC curing	Immediate	Temperature change	Not explicitly stated in extractable article text	LCU influenced intrapulpal temperat	Modified QUIN	High risk

												ure rise		
Chang et al. (2010) [24]	In vitro	10^6 cells / 10-cm dish	Dental pulp cells	None	Bis-GMA	Concentration groups	Not applicable (direct cell model)	24 h exposure	ROS / apoptosis / cell-cycle	MTT assay, ROS detection, cell-cycle analysis, apoptosis assays, RT-PCR and protein analysis	Bis-GMA altered oxidative-stress and apoptosis-related pathways across concentration-dependent exposure groups	Bis-GMA induced oxidative stress, apoptosis-related changes, and cell-cycle effects, indicating that resin monomers may directly compromise pulp-cell homeostasis.	Modified QUIN	High risk
Hadjichristou et al. (2021) [32]	In vitro	n = 36 for Breeze and n = 36 for SpeedCEM Plus in direct-contact PCR experiments; n = 30 per cement in other assays; n = 6	Vascularized dentin-pulp tissue-engineered analogue	None	Breeze; SpeedCEM Plus	Two self-adhesive resin cements	Simulated dentin-pulp interface	Self-curing with optional light cure	24 h and 72 h	Cytocompatibility assay	C1 increased viability to 158 ± 3% at 72 h; C2 reduced viability by about 15–20% at 24 h with recovery by 72 h	Materials differed in biocompatibility	Modified QUIN	High risk

		replicates in 2D tests												
Hiraishi et al. (2003) [17]	In vitro / ex vivo	Not explicitly stated in article text recovered by OCR	Bovine dentine surface model	None	Luting cements including zinc phosphate cement	Material groups	Bovine dentine barrier; 0.5–1.5 mm dentin and smear-layer conditions evaluated	Application on bovine dentine	Immediate setting / pH monitoring period	pH change measurement	pH changed differently among glass-ionomer, zinc phosphate, and zinc polycarboxylate cements	Luting cements differed in acidity and dentin-surface effect	Modified QUIN	High risk
Gonzalez et al. (2018) [29]	In vitro	n=40 samples in medium	Human dental pulp stem cells	None	Glass carbomer; EQUIA Forte Fil; Fuji IX GP Extra; ChemFil Rock; Ketac Molar	Thermo-setting GIC groups	Not applicable (direct cell model)	24 h eluates	Cell viability / mineralization	MTT assay, wound-healing assay, flow cytometry, immunocytochemistry, SEM, and EDX	Forty discs of each material were used for eluate preparation; biologic responses varied by material and dilution	Thermo-setting glass ionomer materials showed non-uniform effects on human dental pulp stem cells, indicating that biologic response remains formulation-dependent even within this	Modified QUIN	High risk

												material class.		
Malkoc et al. (2015) [22]	In vitro	n=10	Bovine dental pulp cells	None	RelyX U200; MaxCem Elite; Multilink Automix and others	Material groups	Not applicable (direct cell model)	Prepared per manufacturers	Not explicitly stated in extractable article text	Real-time cell analysis	Not explicitly stated in extractable article text	Biological response varied among cements	Modified QUIN	High risk
Uzzaman et al. (2005) [40]	Animal in vivo	Monkey teeth; 8–9 teeth/method	Monkey teeth	Hybrid composite resin inlay	Experimental resin-modified glass ionomer luting cement	Different curing methods	Monkey cavities	Manufacturer instructions	Histologic follow-up	Histology	Not explicitly stated in extractable article text	Generally mild pulpal response	SYRCLE	High risk
Bezzon et al. (2015) [42]	Animal in vivo	60 canine premolars	Dog teeth	Deep Class V cavities restored with amalgam	RelyX Unicem; Multilink; zinc phosphate control; gutta-percha control	Material and control groups	0.5-mm dentin	Manufacturer instructions	10 and 90 days	Radiography + histology	No inflammatory cells or bacteria observed	No adverse tissue reactions with RelyX Unicem or Multilink	SYRCLE	High risk
Shimada et al. (2007) [41]	Animal in vivo	8–9 teeth per curing method	Monkey teeth	Composite inlays	Single Bond adhesive + dual-cure resin cement	Different curing methods	Prepared cavities in monkey teeth	Etching gel + Single Bond + inlay bonding	Histologic follow-up	Histology / bacterial penetration	Not explicitly stated in extractable article text	Mild to moderate response influenced by sealing quality	SYRCLE	High risk
Jokstad et al. (2004)	RCT	20 patients / 39	Human vital abutme	Single crowns (metal-	Vitremer	Zinc phosphate	Not explicitly	Split-mouth randomiz	80–104 months	Clinical exam / CDA criteria /	Survival 89% at 102	No long-term difference	Cochrane RoB 2	Low risk

[47]		crown pairs	nt teeth	ceramic and Procera)		cement	stated in extractable article text	ed		sensitivity	months; 96% for no recementation or loss of vitality	between cements		
Kozmacs et al. (2017) [46]	RCT	20 participants / 40 metal-ceramic crowns	Human vital abutment teeth	Metal-ceramic crowns	RelyX Unicem	Zinc phosphate cement	Not explicitly stated in extractable article text	Split-mouth randomized	5 years	VAS + yes/no sensitivity	No significant differences between groups	Comparable long-term hypersensitivity outcomes	Cochrane RoB 2	Some concerns
Saad et al. (2010) [45]	Clinical trial	20 patients / 50 abutments	Human vital abutment teeth	PFM fixed partial dentures	Breeze Self-Adhesive Resin Cement; RelyX Unicem Self-Adhesive Universal Resin Cement	RelyX ARC Adhesive Resin Cement	Not explicitly stated in extractable article text	Clinical cementation	24 h, 2, 6, 12 weeks	VAS to cold, air, biting	RelyX ARC scores significantly higher than Breeze and Unicem	Early sensitivity was material-dependent	Cochrane RoB 2	Some concerns
Sensat et al. (2002) [44]	Clinical trial	22 groups of teeth; 66 teeth treated	Hypersensitive root surfaces of selected teeth	Simulated cast restoration interface	Linkmax and its cement	RelyX ARC and negative control	Not explicitly stated in extractable article text	Surface coating protocol	Immediate and 7 days	Cold sensitivity threshold	Linkmax reduced sensitivity by 8.4 °C immediately and 7.0 °C at 1 week; RelyX ARC by	Linkmax gave significant reduction over 1 week	Cochrane RoB 2	Some concerns

											9.4 °C and 4.3 °C			
Vigolo et al. (2007) [43]	Human in vivo	32 premolars in 8 patients	Human premolars	Porcelain inlays	Composite resin cement	Contralateral control tooth	Preparations entirely in enamel at CEJ	Clinical cementation	1 year	Histology	Failure rate 8%; 2 teeth required endodontic treatment in full cohort	No significant long-term pulpal damage attributable to cementation	ROBINS-I	Moderate risk
Cavied es-Bucheli et al. (2013) [2]	Human in vivo	40 pulp samples	Human pulp tissue	Standardized cavities / indirect cementation context	Vitremer; zinc phosphate cement; adhesive cement	Material groups	Not explicitly stated in extractable article text	Manufacturers' recommendations	Not explicitly stated in extractable article text	Substance P immunoexpression	Not explicitly stated in extractable article text	Increased neurogenic inflammatory signaling	ROBINS-I	Moderate risk
de Mendonça et al. (2007) [23]	In vitro	80 round-shaped samples	MDPC-23 odontoblast-like cells	None	Calcium hydroxide; Vitrebond; RelyX Luting; RelyX Unicem	Material groups	Not applicable (direct cell model)	24 h and 7-day extracts	MTT assay	24 h metabolic reduction: 91.52%, 81.14%, 78.17%, 2.64%; 7-day: 91.13%, 87.27%, 79.04%, 10.51%	RelyX Unicem showed the lowest cytopathic effect	Among the tested hard-setting cements, the biologic response differed markedly by material, with RelyX Unicem showing the most favorable	Modified QUIN	High risk

												cytocompatibility profile in this model.		
Behr et al. (2009) [48]	RCT	49 patients / 49 restorations	Human vital abutment teeth	Metal-based fixed partial dentures	RelyX Unicem Aplicap	Zinc phosphate cement (Richter & Hoffmann)	Finishing line in dentin	Random luting; highly viscous glass-ionomer core build-up	Mean 3.16±0.6 years	Clinical examination incl. vitality/percussion	One endodontic treatment in self-adhesive group; no losses or recementations	Clinical performance comparable to zinc phosphate	Cochrane RoB 2	Some concerns

Supplementary Table S2. PRISMA 2020 for Abstracts checklist

Section and Topic	Item #	Checklist item	Reported (Yes/No)
**TITLE**			
Title	1	Identify the report as a systematic review.	Yes
**BACKGROUND**			
Objectives	2	Provide an explicit statement of the main objective(s) or question(s) the review addresses.	Yes
**METHODS**			
Eligibility criteria	3	Specify the inclusion and exclusion criteria for the review.	Yes
Information sources	4	Specify the information sources (e.g. databases, registers) used to identify studies and the date when each was last searched.	Yes
Risk of bias	5	Specify the methods used to assess risk of bias in the included studies.	Yes
Synthesis of results	6	Specify the methods used to present and synthesise results.	Yes

Section and Topic	Item #	Checklist item	Reported (Yes/No)
**RESULTS**			
Included studies	7	Give the total number of included studies and participants and summarise relevant characteristics of studies.	Yes
Synthesis of results	8	Present results for main outcomes, preferably indicating the number of included studies and participants for each. If meta-analysis was done, report the summary estimate and confidence/credible interval. If comparing groups, indicate the direction of the effect (i.e. which group is favoured).	Yes
**DISCUSSION**			
Limitations of evidence	9	Provide a brief summary of the limitations of the evidence included in the review (e.g. study risk of bias, inconsistency and imprecision).	No
Interpretation	10	Provide a general interpretation of the results and important implications.	Yes
**OTHER**			
Funding	11	Specify the primary source of funding for the review.	Yes
Registration	12	Provide the register name and registration number.	Yes

Supplementary Table S3. PRISMA 2020 checklist

Section and Topic	Item #	Checklist item	Location where item is reported
**TITLE**			
Title	1	Identify the report as a systematic review.	1-3
**ABSTRACT**			
Abstract	2	See the PRISMA 2020 for Abstracts checklist.	10-33
**INTRODUCTION**			
Rationale	3	Describe the rationale for the review in the context of existing knowledge.	47-59
Objectives	4	Provide an explicit statement of the objective(s) or question(s) the review addresses.	58-66
**METHODS**			
Eligibility criteria	5	Specify the inclusion and exclusion criteria for the review and how studies were grouped for the syntheses.	93-113, 178-

Section and Topic	Item #	Checklist item	Location where item is reported
			188
Information sources	6	Specify all databases, registers, websites, organisations, reference lists and other sources searched or consulted to identify studies. Specify the date when each source was last searched or consulted.	115-120
Search strategy	7	Present the full search strategies for all databases, registers and websites, including any filters and limits used.	122-133
Selection process	8	Specify the methods used to decide whether a study met the inclusion criteria of the review, including how many reviewers screened each record and each report retrieved, whether they worked independently, and if applicable, details of automation tools used in the process.	135-139
Data collection process	9	Specify the methods used to collect data from reports, including how many reviewers collected data from each report, whether they worked independently, any processes for obtaining or confirming data from study investigators, and if applicable, details of automation tools used in the process.	141-144
Data items	10a	List and define all outcomes for which data were sought. Specify whether all results that were compatible with each outcome domain in each study were sought (e.g. for all measures, time points, analyses), and if not, the methods used to decide which results to collect.	146-159
	10b	List and define all other variables for which data were sought (e.g. participant and intervention characteristics, funding sources). Describe any assumptions made about any missing or unclear information.	160-162
Study risk of bias assessment	11	Specify the methods used to assess risk of bias in the included studies, including details of the tool(s) used, how many reviewers assessed each study and whether they worked independently, and if applicable, details of automation tools used in the process.	164-170
Effect measures	12	Specify for each outcome the effect measure(s) (e.g. risk ratio, mean difference) used in the synthesis or presentation of results.	172-176
Synthesis methods	13a	Describe the processes used to decide which studies were eligible for each synthesis (e.g. tabulating the study intervention characteristics and comparing against the planned groups for each synthesis (item #5)).	179-180
	13b	Describe any methods required to prepare the data for presentation or synthesis, such as handling of missing summary statistics, or data conversions.	181
	13c	Describe any methods used to tabulate or visually display results of individual studies and syntheses.	182-183
	13d	Describe any methods used to synthesize results and provide a rationale for the choice(s). If meta-analysis was performed, describe the model(s), method(s) to identify the presence and extent of statistical heterogeneity, and software package(s) used.	184-185
	13e	Describe any methods used to explore possible causes of heterogeneity among study results (e.g. subgroup analysis, meta-regression).	186-187
	13f	Describe any sensitivity analyses conducted to assess robustness of the synthesized results.	188
Reporting bias assessment	14	Describe any methods used to assess risk of bias due to missing results in a synthesis (arising from reporting biases).	190-195
Certainty assessment	15	Describe any methods used to assess certainty (or confidence) in the body of evidence for an outcome.	197-212
**RESULTS**			
Study selection	16a	Describe the results of the search and selection process, from the number of records identified in the search to the number of studies included in the review, ideally using a flow diagram.	215-233
	16b	Cite studies that might appear to meet the inclusion criteria, but which were excluded, and explain why they were excluded.	219-221

Section and Topic	Item #	Checklist item	Location where item is reported
Study characteristics	17	Cite each included study and present its characteristics.	237-253, Supplementary Materials Table S1
Risk of bias in studies	18	Present assessments of risk of bias for each included study.	440-519
Results of individual studies	19	For all outcomes, present, for each study: (a) summary statistics for each group (where appropriate) and (b) an effect estimate and its precision (e.g. confidence/credible interval), ideally using structured tables or plots.	-
Results of syntheses	20a	For each synthesis, briefly summarise the characteristics and risk of bias among contributing studies.	424-431, 441-448
	20b	Present results of all statistical syntheses conducted. If meta-analysis was done, present for each the summary estimate and its precision (e.g. confidence/credible interval) and measures of statistical heterogeneity. If comparing groups, describe the direction of the effect.	432-433
	20c	Present results of all investigations of possible causes of heterogeneity among study results.	433-436
	20d	Present results of all sensitivity analyses conducted to assess the robustness of the synthesized results.	437-438
Reporting biases	21	Present assessments of risk of bias due to missing results (arising from reporting biases) for each synthesis assessed.	440-448
Certainty of evidence	22	Present assessments of certainty (or confidence) in the body of evidence for each outcome assessed.	521-542
**DISCUSSION**			
Discussion	23a	Provide a general interpretation of the results in the context of other evidence.	545-613
	23b	Discuss any limitations of the evidence included in the review.	615-642
	23c	Discuss any limitations of the review processes used.	644-657
	23d	Discuss implications of the results for practice, policy, and future research.	659-675
**OTHER INFORMATION**			
Registration and protocol	24a	Provide registration information for the review, including register name and registration number, or state that the review was not registered.	701-703
	24b	Indicate where the review protocol can be accessed, or state that a protocol was not prepared.	702-703
	24c	Describe and explain any amendments to information provided at registration or in the protocol.	-
Support	25	Describe sources of financial or non-financial support for the review, and the role of the funders or sponsors in the review.	700
Competing interests	26	Declare any competing interests of review authors.	708
Availability of data, code and other	27	Report which of the following are publicly available and where they can be found: template data collection forms; data extracted from included studies; data used for all analyses; analytic code; any other materials used in the review.	706-707

Section and Topic	Item #	Checklist item	Location where item is reported
materials			

References

1. de Souza Costa, C.A.; Hebling, J.; Randall, R.C. Human Pulp Response to Resin Cements Used to Bond Inlay Restorations. Dent. Mater. Off. Publ. Acad. Dent. Mater. 2006, 22, 954–962. https://doi.org/10.1016/j.dental.2005.10.007.

2. Caviedes-Bucheli, J.; Ariza-Garcia, G.; Camelo, P.; Mejia, M.; Ojeda, K.; Azuero-Holguin, M.M.; Abad-Coronel, D.; Munoz, H.R. The Effect of Glass Ionomer and Adhesive Cements on Substance P Expression in Human Dental Pulp. Med. Oral Patol. Oral Cir. Bucal 2013, 18, e896–e901. https://doi.org/10.4317/medoral.19111.

3. Kerezoudi, C.; Gogos, C.; Samanidou, V.; Tziafas, D.; Palaghias, G. Evaluation of Monomer Leaching from a Resin Cement through Dentin by a Novel Model. Dent. Mater. 2016, 32, e297–e305. https://doi.org/10.1016/j.dental.2016.09.027.

12. Alvarez, M.M.P.; de Carvalho, R.G.; Barbosa, S.C.d.A.; Polassi, M.R.; Nascimento, F.D.; D'Alpino, P.H.P.; Tersariol, I.L.d.S. Oxidative Stress Induced by Self-Adhesive Resin Cements Affects Gene Expression, Cellular Proliferation and Mineralization Potential of the MDPC-23 Odontoblast-like Cells. Dent. Mater. 2019, 35, 606–616. https://doi.org/10.1016/j.dental.2019.02.008.

13. D'Alpino, P.H.P.; Moura, G.E.D.d.D.; Barbosa, S.C.d.A.; Marques, L.d.A.; Eberlin, M.N.; Nascimento, F.D.; Tersariol, I.L.d.S. Differential Cytotoxic Effects on Odontoblastic Cells Induced by Self-Adhesive Resin Cements as a Function of the Activation Protocol. Dent. Mater. 2017, 33, 1402–1415. https://doi.org/10.1016/j.dental.2017.09.011.

14. Garcia, L.d.F.R.; Pontes, E.C.V.; Basso, F.G.; Hebling, J.; Costa, C.A.d.S.; Soares, D.G. Transdentinal Cytotoxicity of Resin-Based Luting Cements to Pulp Cells. Clin. Oral Investig. 2016, 20, 1559–1566. https://doi.org/10.1007/s00784-015-1630-1.

15. Kong, N.; Jiang, T.; Zhou, Z.; Fu, J. Cytotoxicity of Polymerized Resin Cements on Human Dental Pulp Cells in Vitro. Dent. Mater. Off. Publ. Acad. Dent. Mater. 2009, 25, 1371–1375. https://doi.org/10.1016/j.dental.2009.06.008.

16. Ülker, H.E.; Hiller, K.A.; Schweikl, H.; Seidenader, C.; Sengun, A.; Schmalz, G. Human and Bovine Pulp-Derived Cell Reactions to Dental Resin Cements. Clin. Oral Investig. 2012, 16, 1571–1578. https://doi.org/10.1007/s00784-011-0657-1.

17. Hiraishi, N.; Kitasako, Y.; Nikaido, T.; Foxton, R.M.; Tagami, J.; Nomura. Acidity of conventional luting cements and their diffusion through bovine dentine. Int. Endod. J. 2003, 36, 622–628. https://doi.org/10.1046/j.1365-2591.2003.00700.x.

18. de Mendonça, A.A.; de Oliveira, C.F.; Hebling, J.; Costa, C.A. Influence of thicknesses of smear layer on the transdentinal cytotoxicity and bond strength of a resin-modified glass-ionomer cement. Braz. Dent. J. 2012, 23, 379–386. https://doi.org/10.1590/s0103-64402012000400012.

19. Schmid-Schwap, M.; Franz, A.; König, F.; Bristela, M.; Lucas, T.; Piehslinger, E.; Watts, D.C.; Schedle, A. Cytotoxicity of Four Categories of Dental Cements. Dent. Mater. 2009, 25, 360–368. https://doi.org/10.1016/j.dental.2008.08.002.

20. Annunziata, M.; Aversa, R.; Apicella, A.; Annunziata, A.; Apicella, D.; Buonaiuto, C.; Guida, L. In Vitro Biological Response to a Light-Cured Composite When Used for Cementation of Composite Inlays. Dent. Mater. 2006, 22, 1081–1085. https://doi.org/10.1016/j.dental.2005.08.009.

21. Nakagawa, K.; Saita, M.; Ikeda, T.; Hirota, M.; Park, W.; Lee, M.C.-I.; Ogawa, T. Biocompatibility of 4-META/MMA-TBB Resin Used as a Dental Luting Agent. J. Prosthet. Dent. 2015, 114, 114–121. https://doi.org/10.1016/j.prosdent.2014.10.016.

22. Malkoç, M.A.; Demir, N.; Şengün, A.; Bozkurt, Ş.B.; Hakki, S.S. Cytotoxicity Evaluation of Luting Resin Cements on Bovine Dental Pulp-Derived Cells (bDPCs) by Real-Time Cell Analysis. Dent. Mater. J. 2015, 34, 154–160. https://doi.org/10.4012/dmj.2014-167.

23. de Mendonça, A.A.M.; Souza, P.P.C.; Hebling, J.; Costa, C.A.d.S. Cytotoxic Effects of Hard-Setting Cements Applied on the Odontoblast Cell Line MDPC-23. Oral Surg. Oral Med. Oral Pathol. Oral Radiol. Endodontol. 2007, 104, E102–E108. https://doi.org/10.1016/j.tripleo.2007.05.017.

24. Chang, M.C.; Chen, L.I.; Chan, C.P.; Lee, J.J.; Wang, T.M.; Yang, T.T.; Lin, P.S.; Lin, H.J.; Chang, H.H.; Jeng, J.H. The Role of Reactive Oxygen Species and Hemeoxygenase-1 Expression in the Cytotoxicity, Cell Cycle Alteration and Apoptosis of Dental Pulp Cells Induced by BisGMA. Biomaterials 2010, 31, 8164–8171. https://doi.org/10.1016/j.biomaterials.2010.07.049.

25. Sun, S.; Wang, G.L.; Huang, Y.; Diwu, H.L.; Luo, Y.C.; Su, J.; Xiao, Y.H. The Effects of 2-Hydroxyethyl Methacrylate on Matrix Metalloproteinases 2 and 9 in Human Pulp Cells and Odontoblast-like Cells in Vitro. Int. Endod. J. 2018, 51, e157–e166. https://doi.org/10.1111/iej.12812.

26. Lee, J.H.; Lee, H.H.; Kim, H.W.; Yu, J.W.; Kim, K.N.; Kim, K.M. Immunomodulatory/Anti-Inflammatory Effect of ZOE-Based Dental Materials. Dent. Mater. 2017, 33, e1–e12. https://doi.org/10.1016/j.dental.2016.09.012.

27. Ersahan, S.; Oktay, E.A.; Sabuncuoglu, F.A.; Karaoglanoglu, S.; Aydın, N.; Suloglu, A.K. Evaluation of the Cytotoxicity of Contemporary Glass-Ionomer Cements on Mouse Fibroblasts and Human Dental Pulp Cells. Eur. Arch. Paediatr. Dent. 2020, 21, 321–328. https://doi.org/10.1007/s40368-019-00481-1.

28. Kanjevac, T.; Milovanovic, M.; Volarevic, V.; Lukic, M.L.; Arsenijevic, N.; Markovic, D.; Zdravkovic, N.; Tesic, Z.; Lukic, A. Cytotoxic Effects of Glass Ionomer Cements on Human Dental Pulp Stem Cells Correlate with Fluoride Release. Med. Chem. Shariqah United Arab Emir. 2012, 8, 40–45. https://doi.org/10.2174/157340612799278351.

29. Collado-González, M.; Pecci-Lloret, M.R.; Tomás-Catalá, C.J.; García-Bernal, D.; Oñate-Sánchez, R.E.; Llena, C.; Forner, L.; Rosa, V.; Rodríguez-Lozano, F.J. Thermo-Setting Glass Ionomer Cements Promote Variable Biological Responses of Human Dental Pulp Stem Cells. Dent. Mater. 2018, 34, 932–943. https://doi.org/10.1016/j.dental.2018.03.015.

30. Galler, K.; Hiller, K.A.; Ettl, T.; Schmalz, G. Selective Influence of Dentin Thickness upon Cytotoxicity of Dentin Contacting Materials. J. Endod. 2005, 31, 396–399. https://doi.org/10.1097/01.don.0000145428.26880.e5.

31. Soares, D.G.; Brito, C.A.; da Silva, R.H.B.T.; Ribeiro, A.P.D.; Hebling, J.; Costa, C.A.d.S. Cytocompatibility of HEMA-Free Resin-Based Luting Cements According to Application Protocols on Dentine Surfaces. Int. Endod. J. 2016, 49, 551–560. https://doi.org/10.1111/iej.12479.

32. Hadjichristou, C.; Papachristou, E.; Vereroudakis, E.; Chatzinikolaidou, M.; About, I.; Koidis, P.; Bakopoulou, A. Biocompati-bility Assessment of Resin-Based Cements on Vascularized Dentin/Pulp Tissue-Engineered Analogues. Dent. Mater. 2021, 37, 914–927. https://doi.org/10.1016/j.dental.2021.02.019.

33. Nocca, G.; Iori, A.; Rossini, C.; Martorana, G.E.; Ciasca, G.; Arcovito, A.; Cordaro, M.; Lupi, A.; Marigo, L. Effects of Barriers on Chemical and Biological Properties of Two Dual Resin Cements. Eur. J. Oral Sci. 2015, 123, 208–214. https://doi.org/10.1111/eos.12178.

34. Josic, U.; Teti, G.; Maravic, T.; Mazzitelli, C.; D'Alessandro, C.; D'Urso, D.; Forte, A.; Mancuso, E.; Falconi, M.; Cadenaro, M.; et al. 87—Cytocompatibility of Universal Resin Cements on Human Dental Pulp Cells. Dent. Mater. [M24.1][ΓA24.2]2023, 41, e42. https://doi.org/10.1016/j.dental.2025.01.101.

35. Moon, H.K.; Han, S.J.; Li, C.Y.; Won, J.E.; Shim, J.S. Cytotoxicity Evaluation of Dental Cements Containing Resin: Effects of the Degree of Conversion on Cellular Responses. J. Prosthodont. Res. 2026, 70, 51–64. https://doi.org/10.2186/jpr.JPR_D_24_00299.

36. Taher, N.M.; Al-Khairallah, Y.; Al-Aujan, S.H.; Ad'dahash, M. The Effect of Different Light-Curing Methods on Temperature Changes of Dual Polymerizing Agents Cemented to Human Dentin. J. Contemp. Dent. Pract. 2008, 9, 57–64.

37. Onisor, I.; Asmussen, E.; Krejci, I. Temperature Rise during Photo-Polymerization for Onlay Luting. Am. J. Dent. 2011, 24, 250–256.

38. Kincses, D.; Jordáki, D.; Szebeni, D.; Kunsági-Máté, S.; Szalma, J.; Lempel, E. Effect of Ceramic and Dentin Thicknesses and Type of Resin-Based Luting Agents on Intrapulpal Temperature Changes during Luting of Ceramic Inlays. Int. J. Mol. Sci. 2023, 24, 5466. https://doi.org/10.3390/ijms24065466.

39. Gerdolle, D.A.; Mortier, E.; Loos-Ayav, C.; Jacquot, B.; Panighi, M.M. In Vitro Evaluation of Microleakage of Indirect Composite Inlays Cemented with Four Luting Agents. J. Prosthet. Dent. 2005, 93, 563–570. https://doi.org/10.1016/j.prosdent.2005.04.004.

40. Uzzaman, M.A.; Shimada, Y.; Seki, Y.; Tagami, J. Pulpal Response to a Hybrid Composite Resin Inlay Bonded with a Newly Developed Resin-Modified Glass Ionomer Luting Cement. Dent. Mater. J. 2005, 24, 178–186. https://doi.org/10.4012/dmj.24.178.

41. Shimada, Y.; Uzzaman, M.A.; Tagami, J.; Tanaka, T.; Nakata, T.; Nakaoki, Y.; Sano, H. Effect of Curing Method of a Dual-Cure Resin Cement on Monkey Pulpal Reaction after Bonding of Tooth-Colored Inlay. Dent. Mater. J. 2007, 26, 122–128. https://doi.org/10.4012/dmj.26.122.

42. Bezzon, O.L.; Rivera, D.S.H.; Silva, R.A.B.; Oliveira, D.S.B.; Silva-herzog, D.; Nelson-filho, P.; Lucisano, M.P.; Silva, L.A.B. Resin Luting Materials: Tissue Response in Dog’s Teeth. Microsc. Res. Tech. 2015, 78, 1098–1103. https://doi.org/10.1002/jemt.22590.

43. Vigolo, P.; Graiff, L.; Mutinelli, S.; Fonzi, F. Porcelain Inlays Cemented with Composite Resin Cement: An in Vivo Investigation of Pulpal Reaction One Year Following Cementation. J. Prosthodont. 2007, 16, 123–128. https://doi.org/10.1111/j.1532-849X.2007.00177.x.

44. Sensat, M.L.; Brackett, W.W.; Meinberg, T.A.; Beatty, M.W. Clinical evaluation of two adhesive composite cements for the suppression of dentinal cold sensitivity. J. Prosthet. Dent. 2002, 88, 50–53.

45. Saad, D.E.D.; Atta, O.; El-Mowafy, O. The Postoperative Sensitivity of Fixed Partial Dentures Cemented with Self-Adhesive Resin Cements A Clinical Study. J. Am. Dent. Assoc. 2010, 141, 1459–1466. https://doi.org/10.14219/jada.archive.2010.0108.

46. Kozmacs, C.; Schaper, K.; Lauer, H.C.; Piwowarczyk, A. Evaluation of Hypersensitivity after the Placement of Metal Ceramic Crowns Cemented with Two Luting Agents: Long-Term Results of a Prospective Clinical Study. J. Prosthet. Dent. 2017, 118, 347–352. https://doi.org/10.1016/j.prosdent.2016.10.020.

47. Jokstad, A. A Split-Mouth Randomized Clinical Trial of Single Crowns Retained with Resin-Modified Glass-Ionomer and Zinc Phosphate Luting Cements. Int. J. Prosthodont. 2004, 17, 411–416.

48. Behr, M.; Rosentritt, M.; Wimmer, J.; Lang, R.; Kolbeck, C.; Bürgers, R.; Handel, G. Self-Adhesive Resin Cement versus Zinc Phosphate Luting Material: A Prospective Clinical Trial Begun 2003. Dent. Mater. 2009, 25, 601–604. https://doi.org/10.1016/j.dental.2008.11.003.
